# 98. A nationwide intervention to improve pneumococcal vaccination rate among immunocompromised individuals

**DOI:** 10.1093/ofid/ofac492.176

**Published:** 2022-12-15

**Authors:** Shirley Shapiro Ben David, Orna Shamai-Lubovitz, Vered Mourad, Iris Goren, Erica Cohen Iunger, Tamar Alcalay, Angela Irony, Shira Greenfeld, Limor Adler, Amos Cahan

**Affiliations:** Maccabi Healthcare Services, Tel Aviv, Tel Aviv, Israel; Maccabi Healthcare Services, Tel Aviv, Tel Aviv, Israel; Maccabi Healthcare Services, Tel Aviv, Tel Aviv, Israel; Maccabi Healthcare Services, Tel Aviv, Tel Aviv, Israel; Maccabi Healthcare Services, Tel Aviv, Tel Aviv, Israel; Maccabi Healthcare Services, Tel Aviv, Tel Aviv, Israel; Maccabi Healthcare Services, Tel Aviv, Tel Aviv, Israel; Maccabi Healthcare Services, Tel Aviv, Tel Aviv, Israel; Maccabi Healthcare Services, Tel Aviv, Tel Aviv, Israel; Maccabi Healthcare Services, Tel Aviv, Tel Aviv, Israel

## Abstract

**Background:**

Immunocompromised individuals (ICI) are at high risk for infections, some of which are preventable by vaccines. The Israeli MOH recommends PCV13 and PPSV23 vaccines for ICI, but vaccine coverage is suboptimal. The aim of this study was to assess the effectiveness of a project to improve pneumococcal vaccination (PV) rate among ICI in outpatient settings.

**Methods:**

An automated validated, population-based registry of patients with ICI was developed in an Israeli health organization, Maccabi Healthcare Services, serving over 2.5 million members. Included in the registry were patients aged 18 and above, receiving immunosuppressive therapy (IT); patients living with HIV (PLWH); solid organ and bone marrow transplant recipients (TR); patients with advanced kidney disease (AKD); and patients with asplenia. Based on the registry and the Israeli MOH vaccination guidelines, a nationwide quality improvement project aimed at improving PV was implemented which began in October 2019. As part of the project, ICI were waived the need for preapproval for PCV13. During an eligible patient visit, physicians and nurses were prompted with an EHR alert reminding them to consider providing pneumococcal vaccine. In addition, eligible patients were invited via their patient health record (both desktop and mobile) to vaccinate. Vaccination rates during pre- and post-intervention periods were compared using the Chi square test.

**Results:**

A total of 32,297 ICI were identified. Of them, 22,721 were on IT, 1651 PLWH, 1829 were TR ,5267 had AKD, and 1920 were asplenic. During the period October 2019 to October 2021, PCV13 vaccination rates went up from 12% to 54.1% (p< 0.0001), and PPSV23 vaccination rate improved from 44.7% to 62.6% (P < 0.0001).

**Conclusion:**

Using one of the first real-world automated registries for ICI and implementation of targeted automated patient and provider alerts, markedly improved pneumococcal vaccine uptake was observed in this vulnerable population. Similar interventions may be used to increase the adherence for other vaccines, including COVID-19 vaccines.

**Disclosures:**

**Shirley Shapiro Ben David, MD**, pfizer: Grant/Research Support **Limor Adler, MD**, pfizer: Grant/Research Support.

